# Effect of curcumin on amyloid‐like aggregates generated from methionine‐oxidized apolipoprotein A‐I

**DOI:** 10.1002/2211-5463.12372

**Published:** 2018-01-10

**Authors:** Aparna Krishnamoorthy, Narjes Tavoosi, Gary K. L. Chan, Jianfang Liu, Gang Ren, Giorgio Cavigiolio, Robert O. Ryan

**Affiliations:** ^1^ Department of Nutritional Sciences and Toxicology University of California Berkeley CA USA; ^2^ Children's Hospital Oakland Research Institute CA USA; ^3^ Lawrence Berkeley National Laboratory The Molecular Foundry Berkeley CA USA; ^4^ Department of Biochemistry and Molecular Biology University of Nevada Reno NV USA; ^5^ Present address: Department of Biotechnology College of Science University of Tehran Iran

**Keywords:** amyloid‐like aggregate, Apolipoprotein A‐I, curcumin, electron microscopy, fluorescence spectroscopy, methionine oxidation

## Abstract

Curcumin is a polyphenolic phytonutrient that has antineurodegenerative properties. In this study, we investigated the anti‐amyloidogenic properties of curcumin. Following incubation with curcumin, intrinsic tryptophan fluorescence emission of apolipoprotein (apo) A‐I was strongly quenched. At the same time, curcumin fluorescence emission was enhanced. The fluorescence emission spectra of curcumin in the presence of amyloid‐like aggregates formed by methionine‐oxidized (ox) apoA‐I varied, depending on whether curcumin was added before, or after, aggregate formation. The impact of curcumin on the structure of the aggregating material was revealed by the lower amount of β‐structure in ox‐apoA‐I amyloid‐like aggregates formed in the presence of curcumin, compared to aggregates formed without curcumin. However, the kinetics of ox‐apoA‐I amyloid‐like aggregate formation was not altered by the presence of curcumin. Moreover, electron microscopy analysis detected no discernable differences in amyloid morphology when ox‐apoA‐I amyloid‐like aggregates were formed in the presence or absence of curcumin. In conclusion, curcumin interacts with apoA‐I and alters the structure of ox‐apoA‐I amyloid‐like aggregates yet does not diminish the propensity of ox‐apoA‐I to form aggregates.

**Abbreviations**
ANS: 1‐anilinonaphthalene 8‐sulfonic acid
Apo: apolipoprotein
DMSO: dimethylsulfoxide
EM: electron microscopy
FTIR: Fourier transform infrared
IR: infrared
ox: oxidized
PBS: phosphate‐buffered saline
RFU: Relative Fluorescence Units
ThT: thioflavin T
WMF: wavelength of maximum fluorescence emission

Curcumin (diferuloylmethane) is a major constituent of rhizomes of the plant, *Curcuma longa*. Numerous studies have revealed that curcumin is bioactive, manifesting antioxidant, antitumor, and antineurodegenerative properties 1, 2, 3. Curcumin is a hydrophobic polyphenol that is poorly soluble in aqueous media. At the same time, curcumin is known to interact with proteins, a property hypothesized to mediate its antineurodegenerative effects 4. Indeed, studies have revealed that curcumin has a broad protein interactome 5, 6, 7. Importantly, upon interaction with amyloidogenic proteins, such as α‐synuclein and amyloid‐β peptide, curcumin undergoes a dramatic blue shift in wavelength of maximum fluorescence emission (WMF) along with a large enhancement in quantum yield 8, 9. This property of curcumin is similar to other fluorescent dyes known to interact with amyloid including 1‐anilinonaphthalene 8‐sulfonic acid (ANS), thioflavin T (ThT), Congo red, and Nile red 10, 11, 12.

Similar to curcumin, ANS has a very low quantum yield in buffer. Interestingly, upon interaction with exposed hydrophobic surfaces on proteins, ANS fluorescence emission dramatically increases 13. When added to a solution containing apolipoprotein (apo) A‐I, ANS manifests a large enhancement in quantum yield and a 35 nm blue shift in WMF 14. When the C‐terminal domain of apoA‐I is deleted (Δ185‐243), however, the observed enhancement in ANS fluorescence intensity is greatly reduced, indicating the C‐terminal region of apoA‐I contains solvent‐exposed hydrophobic dye binding sites 15, 16.

Whereas ANS interacts with hydrophobic surfaces of proteins, ThT binds more specifically to β‐sheets that are typical of amyloid structures. For instance, a large enhancement in ThT fluorescence was observed upon incubation with apoA‐I amyloid‐like aggregates 17, 18. Although apoA‐I is not inherently amyloidogenic, it is a component of amyloid deposits found in the low pH, oxidative microenvironment of atherosclerotic lesions 19, 20, 21. Recently, Chan and coworkers reported that myeloperoxidase‐induced oxidation of the three methionine residues present in human apoA‐I greatly increases its propensity to form amyloid‐like aggregates *in vitro* 22. Herein, the effect of curcumin on amyloid‐like aggregate formation by methionine‐oxidized apoA‐I was investigated.

## Materials and methods

### Materials

Curcumin was obtained from Cayman Chemical (Ann Arbor, MI, USA) and used without further purification. A 4 mg·mL^−1^ stock solution of curcumin was prepared in dimethylsulfoxide (DMSO). A standard curve generated by serial dilutions of the stock solution was used to determine curcumin concentrations in unknown samples by absorbance at 430 nm. Recombinant human apoA‐I was expressed in *Escherichia coli*, isolated, and the N‐terminal His‐tag removed as described previously 22. Protein concentration was determined by the bicinchoninic acid assay (Pierce Chemical Co., ThermoFisher, www.thermofisher.com) using bovine serum albumin as standard. ThT was purchased from AnaSpec (Fremont, CA, USA).

### ApoA‐I methionine oxidation

Isolated recombinant apoA‐I was incubated overnight at 37 °C in 10 mm sodium phosphate, pH 7.5, 100 μm diethylene triamine pentaacetic acid, 100 mm NaCl, and H_2_O_2_ (1000 : 1 H_2_O_2_ : apoA‐I mol : mol ratio). The concentration of H_2_O_2_ was determined spectrophotometrically (ε_240_ = 39.4 m ^−1^ cm^−1^). Under these conditions, the three methionines in apoA‐I are fully oxidized (ox‐apoA‐I), with no significant modification of other residues 22. Absorbance spectroscopy was performed on a Shimadzu UV‐1800 spectrophotometer.

### Formation of amyloid‐like aggregates

Ox‐apoA‐I (20 μm) was incubated in the presence and absence of curcumin (50 μm) in fabricated glass 1.5 mL microcentrifuge tubes at 37 °C with continuous shaking for 1–5 days (fibrillation incubation).

### Fluorescence spectroscopy

**Figure 1:**
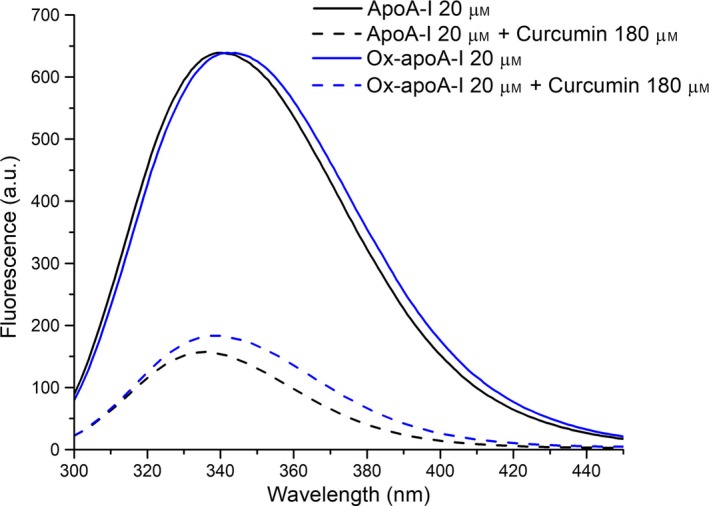
Effect of curcumin on apoA‐I tryptophan fluorescence emission. Samples containing nonoxidized (black lines) or ox‐apoA‐I (blue lines) (20 μm) in PBS alone (solid lines) or PBS plus 180 μm curcumin (dashed lines) were excited at 280 nm and fluorescence emission recorded from 300 to 450 nm.

**Figure 2:**
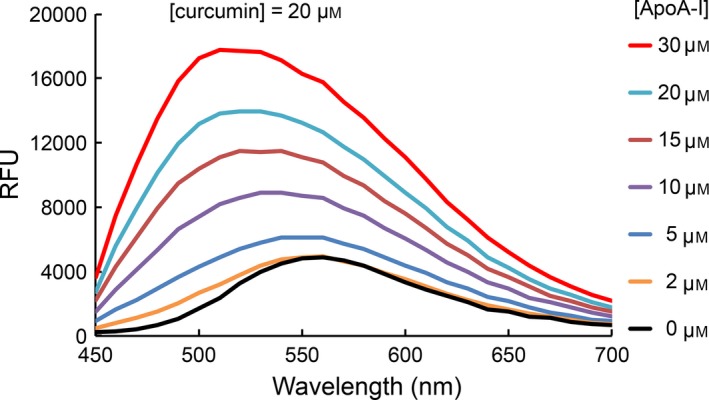
Effect of apoA‐I on the fluorescence emission properties of curcumin. Fluorescence emission spectra of curcumin (20 μm) in PBS were recorded in the presence of increasing concentrations of apoA‐I (0–30 μm). Samples were excited at 420 nm and fluorescence emission recorded between 450 and 700 nm. RFU, Relative Fluorescence Units.

**Figure 3:**
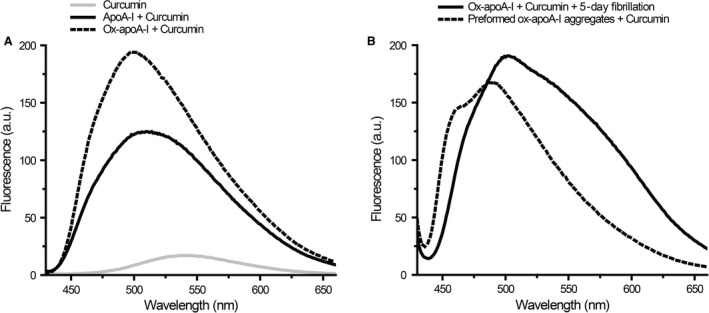
Effect of ox‐apoA‐I physical state on the fluorescence emission properties of curcumin. (Panel A) Fluorescence emission spectra of curcumin in PBS (gray line), curcumin plus nonoxidized apoA‐I (solid black line), and curcumin plus ox‐apoA‐I (dashed line). Curcumin and protein concentrations were 50 and 20 μm, respectively. (Panel B) Ox‐apoA‐I (20 μm) was incubated for 5 days under fibrillation conditions in the absence or in the presence of curcumin (50 μm). Solid black line: curcumin present during fibrillation incubation. Dashed line: curcumin added to amyloid‐like aggregates formed after 5‐day incubation of ox‐apoA‐I under fibrillation conditions. Samples were excited at 420 nm and curcumin fluorescence emission recorded from 425 to 655 nm.

Spectra were obtained on a Horiba Jobin‐Yvon FluoroMax‐4 luminescence spectrometer (Figs 1, 3, 5) and a BioTek Synergy H1 Hybrid Multi‐Mode Reader (Fig. 2). Tryptophan/tyrosine residues in apoA‐I were excited at 280 nm and emission recorded from 300 to 450 nm (2.5 nm slit width for both excitation and emission monochronomators). Curcumin fluorescence emission was recorded from 430 to 600 nm (2.5 nm slit width) following excitation at 420 nm. For kinetics of formation of amyloid‐like aggregates, ThT fluorescence emission spectra were collected by single time‐point dilutions as previously described 22. At indicated time points, an aliquot of the fibrillation mixture (20 μm apoA‐I) was rapidly diluted with stock ThT (500 μm in 50 mm sodium phosphate, pH 7.4) to a final concentration of 18 μm ThT and 80 μg·mL^−1^ apoA‐I (2.84 μm). ThT fluorescence spectra were collected between 460 and 550 nm, with excitation wavelength of 450 nm and 2.5 and 5.0 nm slit widths for the excitation and emission monochronomators, respectively. To construct ThT kinetic curves, the intensity of ThT fluorescence emission at WMF was plotted against time. Experimental replications were obtained by repeating the full ThT kinetics experiment with new samples. Normalized mean ThT fluorescence values at each time point and standard error of the mean (SEM) from at least three independent experiments are reported. Normalized fluorescence emission values were obtained by dividing fluorescence at each time point by the minimum fluorescence emission value (across different independent experiments) recorded at T0 (the time point just before starting the fibrillation incubation).

### Light scattering measurements

Right‐angle light scattering of samples incubated under fibrillation conditions was measured on the Horiba Jobin‐Yvon FluoroMax‐4 spectrometer using the same single time‐point dilution method described in the previous paragraph, with the exception that no ThT was added to the diluted samples. Excitation and emission wavelengths were set at 600 nm, with 2.5 nm slit width. Normalized mean scattering values at each time point and SEM from at least three independent experiments are reported. Normalized scattering values were obtained by dividing scattering at each time point by the minimum scattering value (across different independent experiments) recorded at T0 (time point just before starting the fibrillation incubation).

### Fourier transform infrared (FTIR) spectroscopy

FTIR analysis was performed on a Direct Detect^™^ spectrometer (Merck Millipore, Burlington, MA, USA), as described before 22. At each time pint, 2 μL of incubation mixture was spotted on the Millipore Direct Detect^™^ card. The membrane was then dried and the FTIR spectrum collected. Under these conditions, the FTIR spectrum is representative of the whole sample (soluble + insoluble fractions). Direct comparison of spectral features, such as the β‐signal band visible at about 1622 cm^−1^, provides a direct estimate of the amount of the specific structural element (e.g., β‐structure) in a complex sample.

### Electron microscopy (EM)

Amyloid‐like aggregates were generated by mechanical agitation of ox‐apoA‐I in the presence and absence of curcumin and processed for optimized negative staining as described 23, 24, 25. In brief, the sample was diluted to 60 μg·mL^−1^ protein in Dulbecco's PBS. An aliquot (~ 4 μL) of the sample was placed on a glow‐discharged thin‐carbon‐coated 200‐mesh copper grid (CF200‐Cu, Electron Microscopy Sciences, Hatfield, PA, USA and CU‐200CN, Pacific Grid‐Tech, San Francisco, CA, USA). After ~ 1 min, excess solution was blotted with filter paper, and the grid was washed with water, stained with 1% (w/v) uranyl formate, and dried under a stream of N_2_ gas. Negatively stained specimens were examined on a Zeiss Libra 120 Plus TEM (Carl Zeiss NTS, Oberkochen, Germany) operating at 120 kV with 20 eV in‐column energy filtering, at room temperature. Micrographs were acquired by a Gatan UltraScan 4Kx4K CCD at 80 000× magnification (each pixel corresponding to 1.48 Å) under near Scherzer focus (0.1 μm) and defocus of 0.4 μm. The contrast transfer function parameters of each micrograph were determined and corrected.

## Results

### Effect of curcumin on the intrinsic fluorescence emission properties of apoA‐I

ApoA‐I was employed in experiments to test the hypothesis that curcumin possesses intrinsic anti‐amyloidogenic properties. Initially, interaction between curcumin and nonoxidized apoA‐I was studied by fluorescence emission spectroscopy (excited at 280 nm). When a ninefold molar excess of curcumin was added to a sample of apoA‐I, significant quenching of apoA‐I intrinsic fluorescence emission was observed (Fig. 1). In control experiments, neither curcumin nor the DMSO vehicle, at the same concentrations used for the experiments in Fig. 1, displayed any significant fluorescence emission when excited at 280 nm (data not shown). The results obtained suggest that curcumin interacts with isolated apoA‐I. This interaction was preserved when the three methionine residues of apoA‐I were oxidized to methionine sulfoxides (ox‐apoA‐I). Quenching of the intrinsic fluorescence emission of ox‐apoA‐I by curcumin was similar to the curcumin‐dependent fluorescence quenching observed in nonoxidized apoA‐I (Fig. 1).

### Effect of apoA‐I on the fluorescence emission properties of curcumin

Given the ability of curcumin to quench apoA‐I intrinsic fluorescence emission and complementary effects of apoA‐I on the fluorescence emission properties of related dyes 14, 15, the effect of apoA‐I on the fluorescence emission of curcumin was investigated. In the absence of apoA‐I, the fluorescence emission intensity of curcumin (20 μm, excitation 420 nm) was very low, with a WMF of ~ 560 nm. However, when a fixed amount of curcumin (20 μm) was incubated with increasing concentrations of apoA‐I, significant apoA‐I concentration‐dependent WMF blue shift (up to 50 nm) and increase in curcumin fluorescence emission quantum yield were observed (Fig. 2). Taken together, these results indicate that apoA‐I interacts with curcumin and that fluorescence spectroscopy can be used to detect this interaction.

### Effect of ox‐apoA‐I physical state on the fluorescence emission properties of curcumin

Whereas nonoxidized apoA‐I is generally resistant to aggregation/amyloid formation, oxidation of its three methionine residues confers amyloidogenic properties to the protein. Ox‐apoA‐I (pre‐amyloid state) and nonoxidized apoA‐I had distinct effects on the fluorescence emission properties of curcumin. Compared to nonoxidized apoA‐I, ox‐apoA‐I induced a larger enhancement in curcumin fluorescence emission quantum yield and a larger blue shift in WMF (~ 495 nm, Fig. 3A).

To evaluate the effect of formation of amyloid‐like aggregates using the spectral properties of curcumin, ox‐apoA‐I was incubated under fibrillation conditions (mechanical agitation at pH 6.0) in the presence of the polyphenol. Following a 5‐day incubation, the fluorescence emission spectrum of curcumin featured a main peak centered at ~ 510 nm, accompanied by a broad spectral component at longer wavelength (~ 550 nm, Fig. 3B).

In a separate experiment, curcumin was added to amyloid‐like aggregates formed by ox‐apoA‐I upon 5‐day incubation under fibrillation conditions in the absence of curcumin. Under these conditions, the fluorescence emission spectrum of curcumin consisted of a main peak centered at 495 nm (similar to the curcumin/ox‐apoA‐I sample in panel A) as well as a substantial spectral shoulder at shorter wavelength (~ 465 nm, Fig. 3B).

Thus, when present during fibrillation incubation of ox‐apoA‐I, the fluorescence emission spectrum of curcumin manifests different properties from those observed upon interaction of the polyphenol with preformed ox‐apoA‐I aggregates. Such differences indicate that specific interactions occur between curcumin and the growing amyloid‐like aggregates. To assess whether curcumin may have affected the amount of amyloid formed during the incubation, after 5‐day incubation of ox‐apoA‐I under fibrillation conditions, samples were pelleted and the protein concentration in the supernatant measured. Compared to the initial protein concentration, under each incubation conditions (presence and absence of curcumin), the protein concentration in the supernatant was reduced to about 90%, indicating that a large majority of ox‐apoA‐I had formed aggregates (data not shown).

### FTIR analysis of ox‐apoA‐I aggregates formed in the presence and absence of curcumin

**Figure 4:**
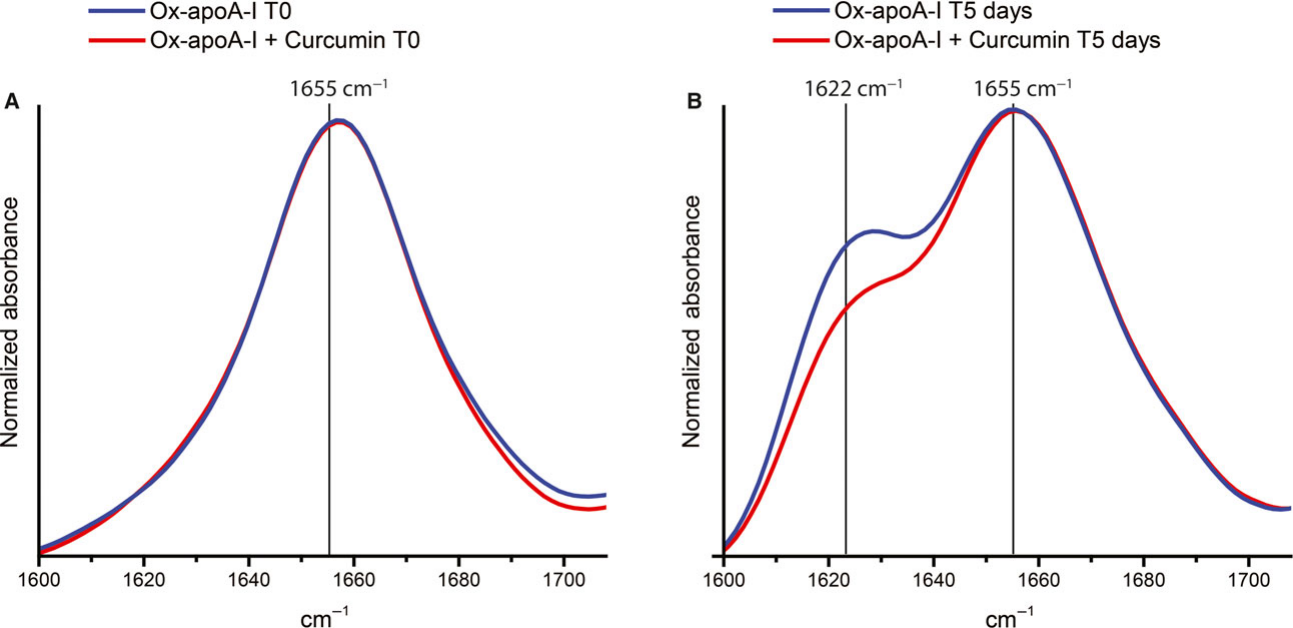
FTIR analysis. Fibrillation incubation mixtures containing ox‐apoA‐I only (20 μm) (blue) or ox‐apoA‐I (20 μm) plus curcumin (50 μm) (red) were analyzed by FTIR before incubation under fibrillation conditions (T0, panel A) and after 5‐day incubation under fibrillation conditions (T5 days, panel B).

The structure of lipid‐free apoA‐I is predominantly α‐helical and random‐coil 26. Methionine oxidation *per se* does not produce a measurable change in the overall structure of apoA‐I, as previously established by circular dichroism, FTIR, and two‐dimensional infrared (IR) spectroscopy 22. The absence of β‐structure in ox‐apoA‐I, both in the presence or in the absence of curcumin, is illustrated by the single peak at about 1655 cm^−1^ in the amide I region of the FTIR spectra in Fig. 4A, which were collected immediately after preparation of the samples and before starting fibrillation incubation.

To investigate whether curcumin affects the structural changes that occur in ox‐apoA‐I upon formation of amyloid‐like aggregates, we compared FTIR spectra of ox‐apoA‐I incubation mixtures upon 5‐day incubation in the presence or in the absence of curcumin. The new peak visible at about 1622 cm^−1^ indicates formation of a significant amount of β‐structures under both incubation conditions (Fig. 4B). This IR β‐signal is characteristic of amyloid‐like structures 27. Remarkably, the amount of β‐structure in ox‐apoA‐I formed in the presence of curcumin (red spectrum) was significantly lower than that formed in the absence of the polyphenol (blue spectrum) (Fig. 4B). These results support the hypothesis that curcumin alters structural features of the amyloid‐like aggregates formed by ox‐apoA‐I.

### Effect of curcumin on the kinetics of formation of amyloid‐like aggregates by ox‐apoA‐I

**Figure 5:**
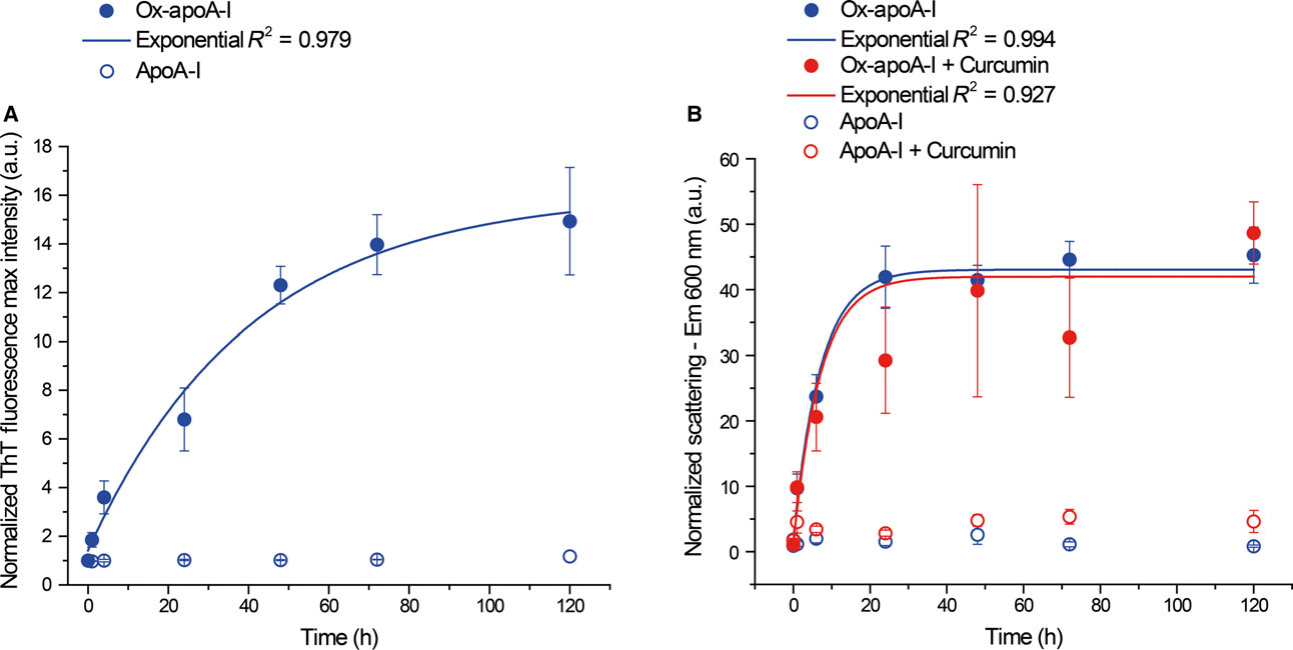
Kinetics of amyloid‐like aggregate formation by ox‐apoA‐I. Nonoxidized (empty circles) or ox‐apoA‐I (filled circles) (20 μm) was incubated under fibrillation conditions in the absence (blue) or presence (red) of curcumin (50 μm). ThT fluorescence emission spectra (panel A) and light scattering (panel B) were recorded at the indicated time points, and kinetics plots were constructed as described in Materials and methods. Normalized mean ThT fluorescence (panel A) and scattering (panel B) values at each time point ± SEM from at least three independent experiments are reported. Solid lines represent a best fit of the experimental mean values by exponential curves.

A classical technique for the evaluation of amyloid formation utilizes ThT as amyloid‐specific dye that fluoresces upon binding to amyloids. Kinetics of amyloid formation can be constructed by recording the time‐dependent increase in ThT fluorescence upon amyloid formation (see Materials and methods) 22. Incubation of ox‐apoA‐I (20 μm apoA‐I) in fibrillation conditions produced a rapid increase in ThT fluorescence, with maximum levels reached between 48‐ and 72‐h incubation (Fig. 5A). ThT fluorescence could not be used to detect amyloid formation in the presence of curcumin because curcumin's fluorescence is also strongly excited at the excitation wavelength of ThT (450 nm). To compare the kinetics of ox‐apoA‐I aggregation in the presence and in the absence of curcumin, light scattering intensity measurements were used as an alternative method. Although light scattering only measures the time‐dependent formation of insoluble aggregates and is not a specific indicator of amyloid formation, the kinetics of aggregate formation, as measured by light scattering (Fig. 5B, blue), were similar to the kinetics of amyloid formation measured by ThT fluorescence (Fig. 5A). Thus, under conditions where classical ThT fluorescence methods are not feasible (i.e., curcumin interference), light scattering served as surrogate technique to measure rates of protein aggregation, a process that precedes and indirectly informs on the kinetics of amyloid formation 28.

When measured by light scattering, no significant differences in kinetics of aggregate formation in the presence (50 μm) or absence of curcumin were detected (Fig. 5B, red and blue, respectively). It is important to note that no significant changes in ThT fluorescence or light scattering were detected when nonoxidized apoA‐I was incubated under the same conditions. Taken together, these results strongly suggest curcumin does not affect the propensity of ox‐apoA‐I to form amyloid‐like aggregates.

### Effect of curcumin on the morphology of ox‐apoA‐I amyloid‐like aggregates

**Figure 6:**
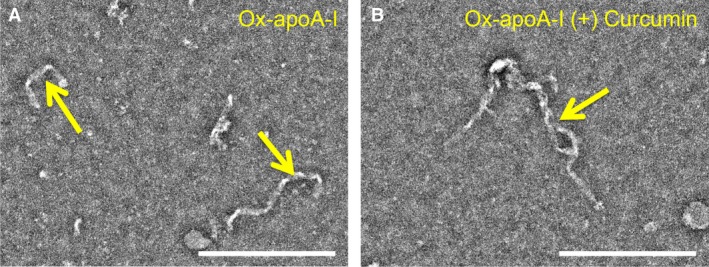
Effect of curcumin on ox‐apoA‐I amyloid‐like aggregate morphology. Ox‐apoA‐I (20 μm) was incubated under fibrillation conditions for 24 h in the absence or in the presence of curcumin (50 μm) as described in Materials and methods. Samples were negatively stained with uranyl formate and imaged by electron microscopy. Representative micrographs of ox‐apoA‐I amyloid‐like aggregates generated in the absence (A) and in the presence (B) of curcumin are reported. Arrows indicate amyloid‐like aggregates. Scale bar = 200 nm.

To evaluate whether the structural differences observed in ox‐apoA‐I aggregates formed in the presence or in the absence of curcumin reflect in the formation of morphologically distinct amyloid‐like aggregates, samples obtained under the two different conditions were imaged by EM (Fig. 6). Upon close inspection, no discernable differences were observed in terms of the amount, morphology, or length of amyloid‐like aggregates present in the two samples.

## Discussion

Fluorescent dyes, including ThT, Congo red, and Nile red, undergo a characteristic enhancement in fluorescence emission upon interaction with amyloid. Furthermore, Congo red emits a green birefringence under polarized light upon binding amyloid fibrils. Given these properties, ThT and Congo red are commonly used to detect amyloid plaque deposits in postmortem tissue samples 29, 30, 31, 32, 33. At the same time, fluorescent dyes such as ANS are valuable reagents for *in vitro* detection of solvent‐exposed hydrophobic sites/conformational changes in proteins. The polyphenolic phytonutrient curcumin shares many of these fluorescence characteristics yet has also been studied for its neuro‐protective effects 34.

In an effort to investigate potential intrinsic anti‐amyloidogenic properties of curcumin, we selected a protein system capable of transitioning from a soluble to amyloid‐like aggregate state under controlled conditions. Based on previous studies 18, 22, methionine‐oxidized apoA‐I was employed as the model amyloidogenic system. As little has been reported on the interaction between curcumin and apoA‐I, we initially investigated the influence of curcumin on the intrinsic fluorescence emission properties of this apolipoprotein. In studies with nonoxidized apoA‐I and ox‐apoA‐I, it was determined that curcumin is a highly effective fluorescence quenching agent (see Fig. 1). In complementary experiments, as seen in Fig. 2, apoA‐I also had a dramatic effect on the intrinsic fluorescence properties of curcumin, similar to ANS and related fluorescent dyes. Two key features were apparent: an approximate fivefold enhancement in curcumin fluorescence emission quantum yield and a ~ 50 nm blue shift in WMF. A similar, but even more pronounced effect was measured in the case of ox‐apoA‐I (Fig. 3A). Given this evidence of a molecular interaction between curcumin and apoA‐I, both in its nonoxidized and methionine‐oxidized forms, the potential impact of curcumin on amyloid‐like aggregate formation by ox‐apoA‐I was investigated.

Ox‐apoA‐I forms amyloid‐like aggregates upon mechanical agitation, providing a controlled method to induce aggregation and assess the effect of curcumin on the aggregation process. When the fluorescence emission of curcumin was investigated in the presence of ox‐apoA‐I amyloid‐like aggregates, significant differences in the spectral properties of curcumin were observed, depending on whether the polyphenol was introduced before incubating ox‐apoA‐I under fibrillation conditions or added after such incubation (Fig. 3B). These spectral differences indicate a different interaction mode of the polyphenol with the amyloid‐like aggregates depending on whether curcumin interacts only at the surface level of the aggregates or can be incorporated within aggregates. This finding suggests that curcumin, if present during the aggregation process, could alter the structure of the final aggregates by interacting with the soluble intermediates that are the amyloidogenic building blocks. FTIR analysis of the secondary structure of ox‐apoA‐I amyloid‐like aggregates formed in the absence or in the presence of curcumin revealed that the polyphenol impacts the amount of β‐structure formed by ox‐apoA‐I.

Despite this significant effect of curcumin on the structure of ox‐apoA‐I amyloid‐like aggregates, no differences in the kinetics of aggregation were detected as a function of the presence or absence of curcumin. Furthermore, negative‐stain EM analysis of aggregates produced in the two conditions revealed no discernable differences in morphology, size, or number of the amyloid‐like aggregates present in the samples.

Although the relatively rapid *in vitro* amyloid generation protocol employed failed to provide evidence that curcumin possesses anti‐amyloid properties, in terms of kinetics of aggregate formation and morphologies of the final aggregates, the data indicate a clear effect of the polyphenol on the structure of the final ox‐apoA‐I aggregates. It is therefore possible that long‐term consumption of dietary or supplemental curcumin impacts the process of amyloidogenesis *in vivo*. A more refined, perhaps slower, *in vitro* amyloid generation system may be required to fully evaluate this hypothesis.

A key value of curcumin as an intervention molecule is its widespread use as a cooking spice and dietary supplement 34. Given its ubiquitous use, the proposed antineurodegenerative and cardioprotective properties of curcumin continue to be of great interest. This study characterizes the interaction of curcumin with apoA‐I and its amyloid‐like aggregates and provides evidence of the ability of curcumin to alter the structure of ox‐apoA‐I amyloid‐like aggregates. Further investigation is needed to evaluate the potential effect of curcumin on the aggregation properties of ox‐apoA‐I *in vivo*.

## Author contributions

AK and NT conducted fluorescence spectroscopy studies. GKLC and GC performed incubations under fibrillation conditions and analyzed the products. GC conducted thioflavin T and FTIR experiments. JL and GR conducted electron microscopy analysis. ROR conceived and designed the study and, together with AK and GC, wrote the manuscript.
